# CD36 is expressed in a defined subpopulation of neurons in the olfactory epithelium

**DOI:** 10.1038/srep25507

**Published:** 2016-05-05

**Authors:** André Machado Xavier, Raissa Guimarães Ludwig, Maíra Harume Nagai, Tiago Jonas de Almeida, Hebe Mizuno Watanabe, Marcio Yukio Hirata, Tatiana Rosado Rosenstock, Fabio Papes, Bettina Malnic, Isaias Glezer

**Affiliations:** 1 Department of Biochemistry, Escola Paulista de Medicina, Universidade Federal de São Paulo, Brazil; 2 Department of Biochemistry, Instituto de Química, Universidade de São Paulo, Brazil; 3 Department of Genetics and Evolution, Instituto de Biologia, Universidade de Campinas, Brazil; 4 Department of Physiological Science, Santa Casa de São Paulo Medical School, Brazil.

## Abstract

The sensory neurons in the olfactory epithelium (OSNs) are equipped with a large repertoire of olfactory receptors and the associated signal transduction machinery. In addition to the canonical OSNs, which express odorant receptors (ORs), the epithelium contains specialized subpopulations of sensory neurons that can detect specific information from environmental cues and relay it to relevant neuronal circuitries. Here we describe a subpopulation of mature OSNs in the main olfactory epithelium (MOE) which expresses CD36, a multifunctional receptor involved in a series of biological processes, including sensory perception of lipid ligands. The *Cd36* expressing neurons coexpress markers of mature OSNs and are dispersed throughout the MOE. Unlike several ORs analyzed in our study, we found frequent coexpression of the OR *Olfr287* in these neurons, suggesting that only a specific set of ORs may be coexpressed with CD36 in OSNs. We also show that CD36 is expressed in the cilia of OSNs, indicating a possible role in odorant detection. CD36-deficient mice display no signs of gross changes in the organization of the olfactory epithelium, but show impaired preference for a lipid mixture odor. Our results show that CD36-expressing neurons represent a distinct population of OSNs, which may have specific functions in olfaction.

Olfactory sensory neurons (OSNs) in the olfactory epithelium (OE) express different types of olfactory receptors, which participate in the detection of distinct chemosensory stimuli. This information is subsequently transmitted to the olfactory bulb (OB) in the central nervous system^1^,^2^. Mature OSNs express the olfactory marker protein (OMP)^3^ and are functionally and molecularly classified according to the type of olfactory receptor they express, their pattern of axonal projection to olfactory glomeruli in the OB, or presence of specific signal transduction machinery^4^,^5^. Most OSNs in the main olfactory epithelium (MOE) express canonical odorant receptors (ORs), which belong to a large family of over a thousand G-protein coupled receptors^6^ and function in a combinatorial manner to discriminate an enormous number of odorants^7^. Other OSNs in the MOE express instead trace amine-associated receptors (TAARs) or receptor guanylyl cyclase (GC-D). These receptors are involved in the detection of volatile amines^8^,^9^,^10^ and peptide hormones^11^ present in urine, respectively. It has also been shown that GC-D expressing neurons detect CO_2_, which is an important olfactory stimulus for many organisms^12^.

Distinct subpopulations of OSNs in the olfactory epithelium may also differ in the signaling proteins they express. While the canonical OSNs express the olfactory G protein alpha subunit (Gαolf/*Gnal*), adenylyl cyclase III (ACIII/*Adcy3*) and the cyclic nucleotide-gated (*Cnga2*) channel, other subpopulations express members of the transient receptor potential (Trp) channel family or other subtypes of transduction proteins^4^.

CD36, also known as fatty acid transporter (FAT), is a single-chain glycoprotein expressed at the cell surface of several mammalian cells types^13^. Most of the receptor is extracellular; it possesses two transmembrane segments and two short cytoplasmatic portions. Even though its intracellular signaling capacity is apparently limited, CD36 is involved in multiple cellular functions, including lipid binding, lipid uptake and inflammatory signaling^13^,^14^. In addition, CD36 functions in the immune system as a scavenger receptor that cooperates with *Toll*-like receptors (TLRs) to recognize lipids derived from bacterial pathogens^15^.

Moreover, CD36 is known to play several roles in sensory perception. The sensory neuron membrane protein (SNMP), a *D. melanonogaster* CD36 homologue, mediates proper responses to the lipid pheromone cis-vaccenyl acetate (cVA) in a population of sensory olfactory neurons^16^,^17^. In addition, it was shown that CD36 mediates fatty acid detection in taste cells and preference for lipid ingestion in rodents^18^,^19^, suggesting a conserved role for this receptor in lipid sensory recognition.

In previous studies^20^ we showed that the CD36 gene is expressed in discrete regions of the brain that receive input from both the main and accessory olfactory bulbs^21^,^22^,^23^,^24^. Here we show that CD36 is expressed in the cilia of a subpopulation of OSNs in the MOE. These neurons coexpress typical markers for mature olfactory sensory neurons, including proteins involved in the canonical OR signal transduction cascade. We found that some of these CD36 expressing neurons also coexpress *Olfr287*. No coexpression was however observed when other types of ORs were analyzed, indicating that the CD36 expressing neurons may express only a particular subgroup of all possible existing ORs. In addition, we also show that innate preference for lipid odor is impaired in CD36-deficient mice. Altogether, our results suggest that CD36 may play important roles in olfaction.

## Results

### Cd36 gene expression is abundant in the olfactory epithelium

**Figure 1:**
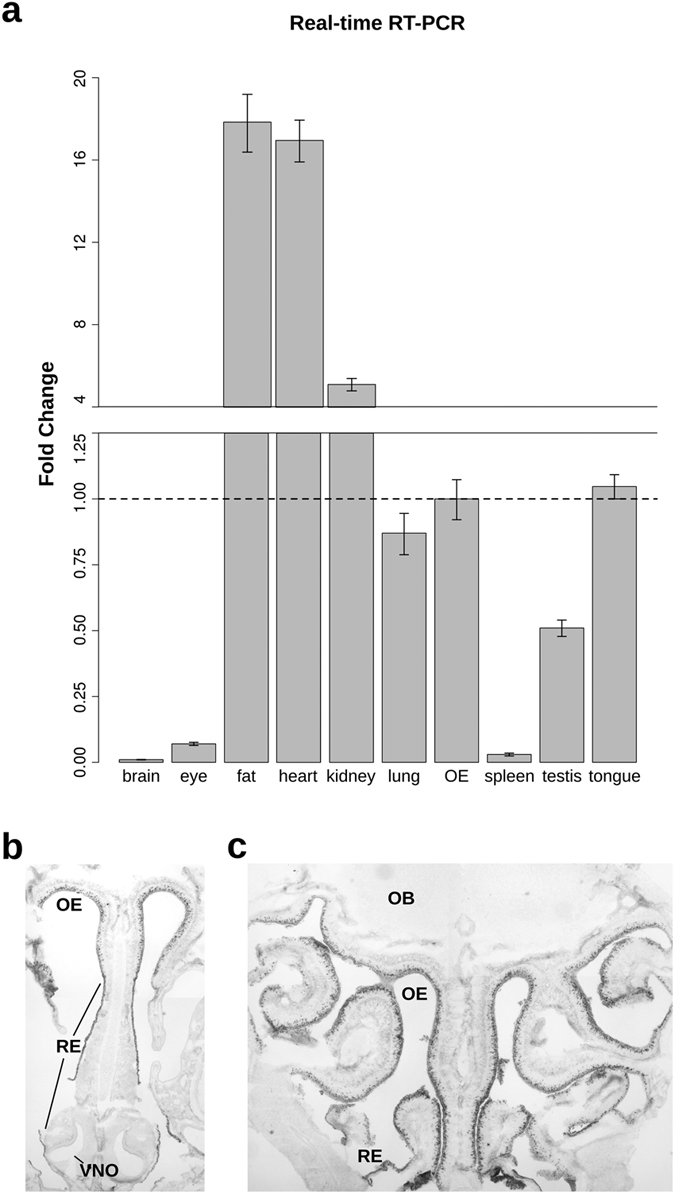
*Cd36* gene is expressed in the olfactory epithelium. (**a**) Real-time RT-PCR analysis of *Cd36* transcript levels normalized against *Actb* housekeeping gene for each tissue, represented as fold-change (OE as calibrator; mean of triplicates ± S.D.). Representative microphotographs show widespread pattern of *Cd36* mRNA *in situ* hybridization labeling in rostral (**b**) and caudal (**c**) OE cryosections from 4-week-old mice. Note the absence of gene expression in the VNO and olfactory bulb. Abbreviations: OB, olfactory bulb; OE, olfactory epithelium; RE, respiratory epithelium; VNO, vomeronasal organ.

In order to evaluate the relative abundance of *Cd36* transcripts, we conducted real-time RT-PCR using various mouse tissues and two different reference genes (Figs 1a and S1). High levels of *Cd36* expression were observed in tissues known to strongly express the gene, such as the heart and adipose tissue (17- fold the expression levels in OE; Fig. 1a), in agreement with the transcript levels originally described for rat tissues^14^. *Cd36* expression in the OE is much higher than in other neural tissues, such as eye or whole brain. Interestingly, *Cd36* expression in the dorsal tongue, implicated in fat intake preference^19^, has not been previously compared to the expression in other tissues. Here we report that *Cd36* expression in the OE is comparable to that in the dorsal tongue, both representing epithelial tissues that perform environmental chemical sensing (Fig. 1a).

*In situ* hybridization experiments revealed that *Cd36* expression is widely distributed throughout the MOE of adult mice, but absent in the vomeronasal organ (VNO; Fig. 1b) or in the OB (Fig. 1c). The respiratory epithelium also stains positive for the *Cd36* message, forming a continuous labeled contour (Fig. 1b). It is remarkable, however, that even though the olfactory epithelium shows intense staining in numerous cells in the neuronal cell layer, only a subset of the cells are stained. These results indicate that *Cd36* expression in the MOE is restricted to a subpopulation of neurons.

**Figure 2:**
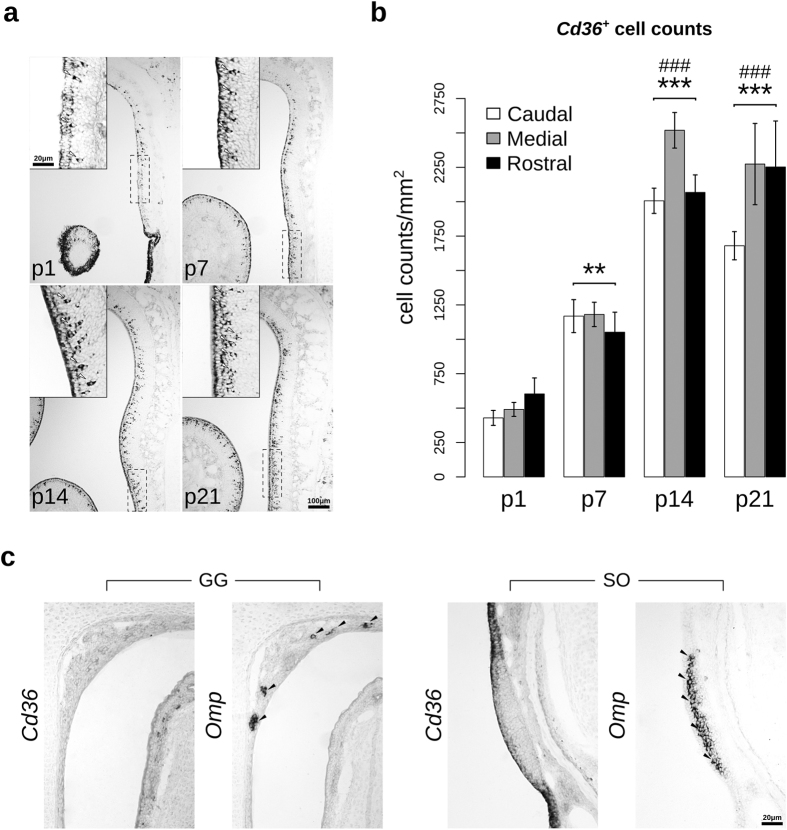
Cellular density of *Cd36* mRNA-positive cells increases with postnatal development of the olfactory epithelium. (**a**) *Cd36* riboprobe signals (white arrowheads) at different postnatal ages (days): p1, p7, p14 and p21. Inserts show higher magnification of the area indicated by dashed rectangles. (**b**) Quantification of cell density (cell counts/OE area; mean ± S.E.M.) showed in ‘**a**’, which was performed excluding the stained apical contour cells (supporting cells; SCs). Two-way ANOVA revealed a highly significant main age effect *F*_(3,24)_ = 73.877, *p* = 2.91 × 10^−12^, while the level of the olfactory epithelium (rostral, medial and caudal) effect was only marginal, *F*_(2,24)_ = 3.367, *p* = 0.0514. (**c**) Absence of *Cd36* transcripts in the Grueneberg ganglion and septal organ, two noncanonical olfactory subsystems that contain *Omp*-positive OSNs (black arrowheads). Abbreviations: GG, Grueneberg ganglion; SO, septal organ. **p < 0.0005 and ***p < 0.00001 compared to p1; ^###^*p* < 0.00001 compared to p7. Scale bars: as indicated.

We next examined the expression of *Cd36* during the postnatal development of the olfactory epithelium. Even though the staining pattern for *Cd36* expression was observed in all developmental stages (Fig. 2a), the number of labeled cells progressively increased until P14, and then stabilized, in all the three levels of the olfactory epithelium measured (rostral, medial and caudal) (Figs 2b and S2). No *Cd36* expression was detected in the septal organ (SO) and Grueneberg ganglion (GG) at any tested age (Fig. 2c).

### Cd36-positive OSNs coexpress OMP, Gαolf, and specific ORs

**Figure 3:**
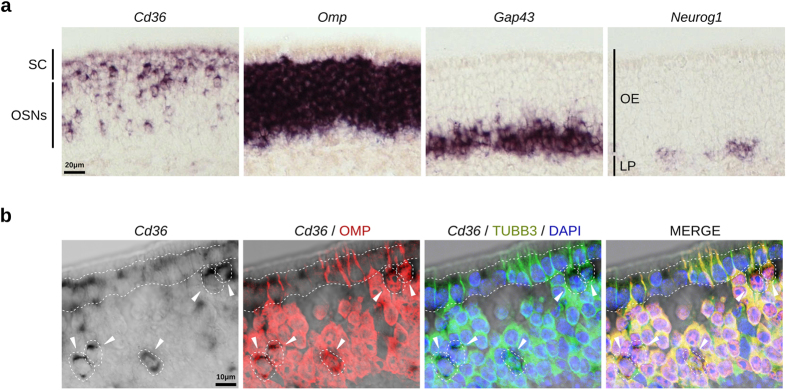
*Cd36* mRNA is expressed in mature OSNs. (**a**) Adjacent sections showing the localization of *Cd36* expressing cells in the olfactory epithelium from 4-week-old mice compared to *Omp* (a marker for mature OSNs), *Gap43* (a marker for immature OSNs), and *Neurog1* (a marker for basal globose cell progenitors) expressing cells. (**b**) Confocal acquisitions showing coexpression of *Cd36* transcripts (chromogenic *in situ* hybridization) with OMP and TUBB3 proteins (immunofluorescence) in the same OSNs (white arrowheads and dashed lines). Sections from 3-month-old mice; nuclei were stained with DAPI. Supporting cell layer is delimited with dashed lines. Abbreviations: OSNs, olfactory sensory neurons; LP, lamina propria; SC, supporting cells. Scale bars are shown.

**Figure 4:**
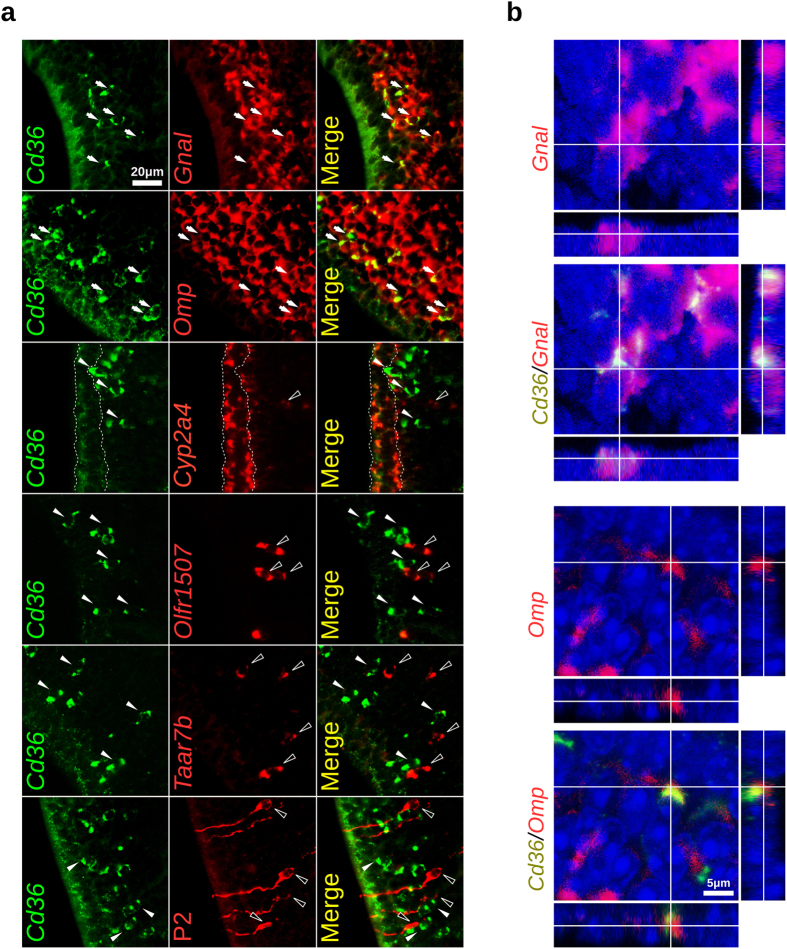
*Cd36* expressing neurons coexpress canonical OSNs transcripts. (**a**) Two-color fluorescent *in situ* hybridization (FISH) with a *Cd36* riboprobe shown in green (white arrowheads) and the other transcripts shown in red (empty arrowheads); arrows indicate coexpression (*Omp* and *Gnal*). While a high frequency of coexpression was observed for the canonical OSNs transcripts, no *Cd36* coexpression was observed for two ORs (*Olfr1507* and P2/*Olfr17*) or members of the *Taar7* subfamily (identified with a *Taar7b* probe) – please refer to Table 1 for details. In the bottom panel, one-color FISH with a *Cd36* riboprobe (green) combined with immunostaining for the GFP protein (red) was performed to identify the P2-IRES-tauGFP expressing neurons. Supporting cells (*Cyp2a4* labeled apical cells; delimited with dashed lines) show weaker labeling for *Cd36* when compared to OSNs. (**b**) Confocal acquisitions with orthogonal projections depicting *Cd36*^+^ OSNs coexpressing *Gnal* and *Omp* transcripts. Nuclei were stained with DAPI (blue). Scale bars are shown.

*Cd36* mRNA hybridization signals in the olfactory epithelium are observed in two different cell layers, in the supporting cell layer (lighter staining) and in the mature OSNs layer (Fig. 3a). It is noteworthy that cells in the OSNs layer show stronger staining than the ones in the supporting cell layer. Combined *in situ* hybridization and immunofluorescence staining shows that CD36 expressing cells coexpress both OMP (mature OSNs) and type III β-tubulin (TUBB3; mature and immature OSNs) (Fig. 3b). In addition, two-color fluorescent *in situ* hybridization experiments revealed that *Cd36* is consistently coexpressed with *Omp* and Gαolf (*Gnal*), the G protein through which ORs signal (Fig. 4a,b, Table 1). In contrast, duct and supporting cells labeled with the *Cyp2a4* riboprobe do not or only weakly express *Cd36* (Fig. 4a). Together, these results show that mature OSNs in the MOE are the main source of the transcript.

Since OR-expressing and TAAR-expressing neurons represent different populations of mature OSNs in the olfactory epithelium, we investigated whether *Cd36*-positive OSNs coexpress some of the members belonging to these two receptor families. We performed two-color fluorescent *in situ* hybridization using *Cd36* riboprobes together with a riboprobe corresponding to an OR gene which is the most highly expressed OR gene in the mouse olfactory epithelium (*Olfr1507*, also known as mOR28)^25^,^26^ or a *Taar7b* probe, which should recognize all of the highly related members of the *Taar7* subfamily (TAARs 7a, 7b, 7d, 7e, and 7f)^9^. Neither *Olfr1507*- nor *Taar7b*-positive OSNs showed coexpression of *Cd36* (Fig. 4a and Table 1). We also used P2-IRES-tauGFP mice, to identify the OSNs expressing the OR P2 (*Olfr17)*^27^ and did not observe coexpression of *Cd36* with GFP in these neurons (Fig. 4a and Table 1). We therefore analyzed a larger number of OR genes. Probes corresponding to additional 11 OR genes were pooled and used in the two-color fluorescent *in situ* hybridization together with the probe for *Cd36* (pool 1: *Olfr124* and *Olfr1509*; pool 2: *Olfr646*, *Olfr1264* and *Olfr1372-ps1*; pool 3: *Olfr78*, *Olfr569*, *Olfr638*, *Olfr691*, *Olfr692*, and *Olfr1512*). As shown in Table 1 and figure S3, no coexpression of *Cd36* with these OR genes was also observed.

**Figure 5:**
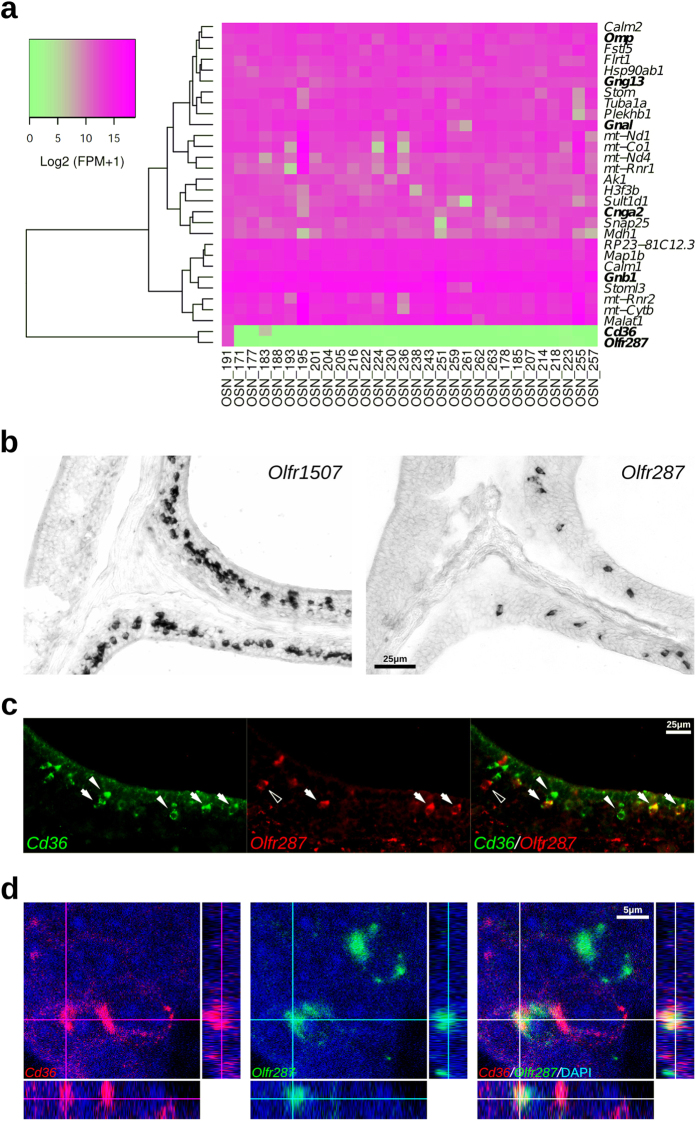
*Olfr287* is frequently coexpressed with *Cd36* transcripts. (**a**) Heatmap from the top thirty expressed genes in a *Cd36*^+^-OSN (OSN #191) according to a single-OSN RNAseq annotation data^28^. *Olfr287* and *Cd36* are only expressed at high levels in this neuron, which also expresses mature OSN marker transcripts (in bold). Transcript levels are expressed as Log_2_ (FPM + 1) (FPM: fragments per million). (**b**) *Olfr287* is expressed in the same expression zone in the OE and less frequently than *Olfr1507*. (**c**) Two-color fluorescent *in situ* hybridization (FISH) with a *Cd36* riboprobe shown in green (white arrowheads) and *Olfr287* shown in red (empty arrowheads); arrows indicate coexpression and arrowheads indicate absence of coexpression in the merged image. (**d**) Confocal acquisition depicting orthogonal projections for coexpression validation; *Cd36* riboprobe shown in red and *Olfr287* shown in green. Scale bars: as indicated.

In a recent study, Saraiva and colleagues used single-cell RNA sequencing (RNAseq) experiments to analyze the transcriptomes of individual OSNs^28^. One of the analyzed neurons (OSN #191) expressed high levels of *Cd36*. Also highly expressed in this same neuron are the typical markers for mature OSNs (*Omp*, *Gng13*, *Gnal*, *Cnga2*, *Gnb1*) and the OR *Olfr287* (Fig. 5a). No relevant expression of TAARs, TrpC2, Trpm5 or GC-D was observed in this neuron. Expression of a second OR gene was also detected in this neuron, although at considerably lower levels than *Olfr287*.

Based on these studies, we next decided to check whether *Cd36*^+^-OSNs coexpress *Olfr287*. *In situ* hybridization experiments showed that *Olfr287*^+^-OSNs are located in the same expression zone as *Olfr1507*^+^-OSNs, but are less numerous (Fig. 5b). Two-color FISH revealed substantial coexpression of this receptor and the *Cd36* transcript (Fig. 5c,d and Table 1). It should be noted that the *Olfr287* probe we used was designed to cover the 3′-untranslated region of the transcript because two other OR genes, *Olfr286* and *Olfr288*, show highly similar coding sequences, and could therefore cross hybridize with the *Olfr287* probe. Using this specific probe we found that ~62% of the *Olfr287*^+^-OSNs coexpress *Cd36*, and ~16% of the *Cd36*^+^-OSNs coexpress *Olfr287* in the same OE zone, indicating that not all *Olfr287*^+^-neurons are positive for *Cd36* and vice-versa. Nevertheless, *Olfr287* coexpression with *Cd36* transcript is a frequent event that was not observed for the other OR genes tested in our experiments.

### CD36 protein localizes to the ciliary layer of the olfactory epithelium

**Figure 6:**
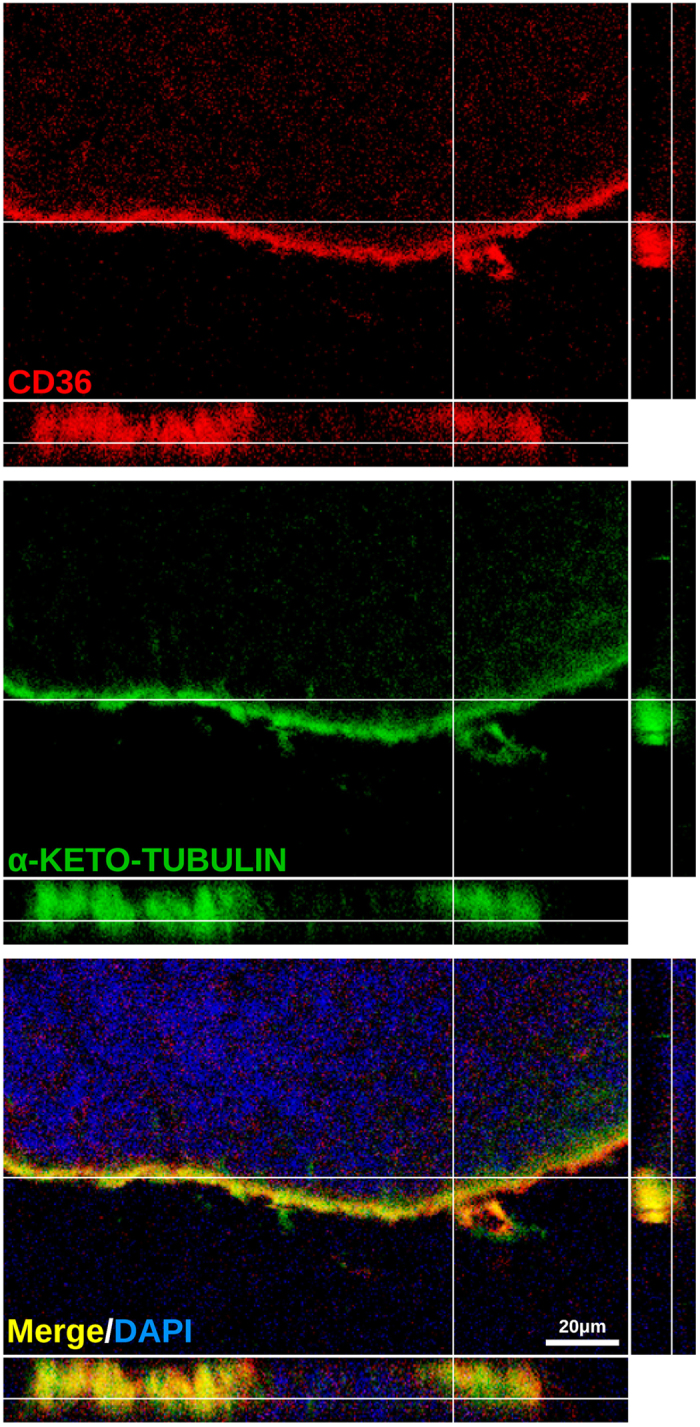
CD36 protein is localized to the ciliary layer in the olfactory epithelium. Confocal images of olfactory epithelium sections immunostained with antibodies against CD36 (red) and acetylated α-tubulin (green, α-keto-tubulin), a protein expressed in the cilia of OSNs. Confocal acquisitions with orthogonal projections show localization of the proteins, indicating that the CD36 protein concentrates in the ciliary layer of the olfactory epithelium. Scale bars: as indicated.

**Figure 7:**
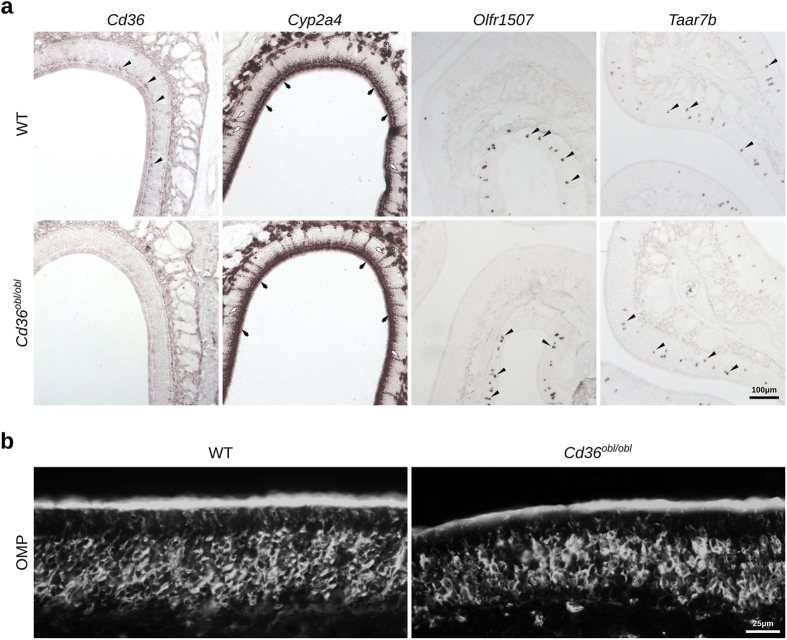
The general organization of the olfactory epithelium from CD36**-deficient mice is indistinguishable from that of wild-type mice. (**a**) *In situ* hybridization was performed on olfactory epithelium sections from age- and sex-matched C57Bl/6 and *Cd36*^*obl/obl*^ mice using riboprobes for *Cd36*, *Olfr1507*, *Taar7b* and *Cyp2a4* (which is normally expressed in supporting cells, duct cells and Bowman’s glands). Except for the reduction of *Cd36* signals in the mutant mouse, expression patterns for the other probes are similar to the ones in the wild type mouse (WT). (**b**) OMP immunofluorescence labeling is equivalent between the two genotypes. Black arrowheads indicate labeled OSNs; black arrows indicate supporting cells; white arrows indicate ducts; asterisks indicate Bowman’s gland. Scale bars are shown.

We subsequently investigated whether CD36 is localized to the cilia of OSNs, the site of odorant detection and signal transduction. Double immunostaining and confocal analysis indicate that CD36 colocalizes with acetylated α-tubulin, a marker for cilia, indicating that the CD36 protein is predominantly expressed in the cilia of the OSNs (Fig. 6 and S4). We also analyzed the general morphology of the OE from the *Cd36*^*obl/obl*^ mutant mice. These mice carry a nonsense mutation that codes for a premature stop codon within the coding region of the *Cd36* gene^15^, and consequently do not express *Cd36* in the olfactory epithelium (Figs 7 and S5). The OE from *Cd36*^*obl/obl*^ mice appears normal, and the density and zonal expression profiles of *Olfr1507* and *Taar7b* are unaltered (Fig. 7). In addition, the general distribution of supporting cells, ducts and Bowman’s gland (which express *Cyp2a4*) and OMP-positive neurons are unaffected in the *Cd36* mutant mice (Fig. 7a,b), and no gross changes in the structure of the olfactory bulb of one-year-old CD36-deficient mice are observed (Fig. S6 and Table S1).

### CD36-deficient mice display altered olfactory behavior upon lipid exposure

**Figure 8:**
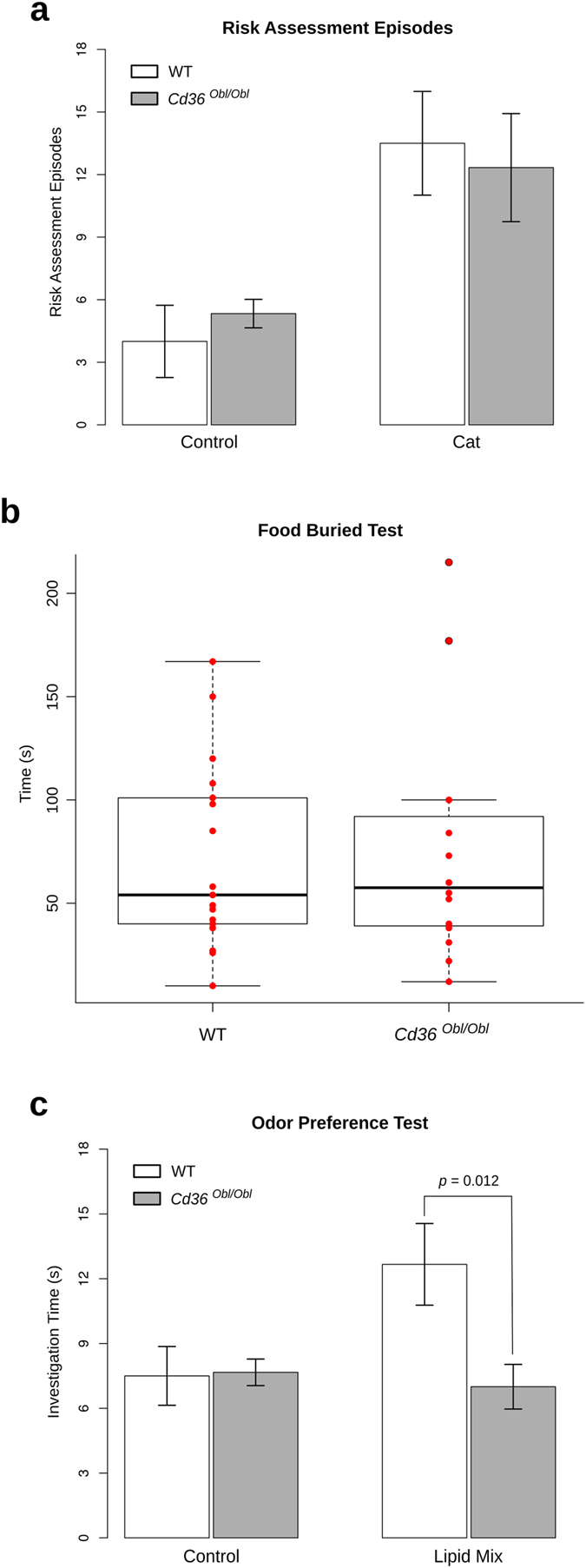
CD36-deficient mice show impaired preference for lipid odorants. (**a**) The number of risk assessments was determined in wild-type and *Cd36*^*obl/obl*^ mutant mice exposed to gauze rubbed over cat fur. CD36-deficient and wild-type mice showed equivalent responses to the predator kairomones (*n* = 6 for all groups except for control gauze exposed wild-type; *n* = 4). (**b**) Food buried test was performed to evaluate the general olfactory ability of *Cd36*^*obl/obl*^ mice. The time required for the animals to find a hidden food pellet was recorded. No statistically significant difference was observed between the two strains. However, innate preference for a filter paper scented with a chemically defined lipid concentrate, evaluated by the investigation time, was lost in *Cd36*^*obl/obl*^mice (*n* = 6/group) (**c**). Statistical analysis was performed using two-way ANOVA (**a**,**c**) and Welch’s test (**b**).

We next analyzed whether olfactory guided behaviors are perturbed in CD36-deficient mice. Given that CD36 is not expressed in the VNO, it is expected that responses to pheromones or kairomones detected by this organ would not be altered in *Cd36*^*obl/obl*^ mice. In fact, we found that defensive behaviors generated upon exposure of mice to predator odors, a response shown to be largely dependent on the VNO^29^, was equivalent in *Cd36*^*obl/obl*^ and wild-type animals (Fig. 8a). In order to assess general olfactory capacity, we conducted the buried food olfactory assay. No significant differences were observed in the performances of wild type and *Cd36*^*obl/obl*^ mice in this test, indicating that the loss of CD36 does not affect general odorant detection (Fig. 8b). In addition, no significant changes were observed between the two genotypes when mice were exposed to amyl acetate in an odorant preference test (Fig. S7).

Since CD36-deficiency does not lead to aberrant OE morphology or general olfactory behavior impairment, we reasoned that only responses to specific odorants that bind to the receptor should be disturbed in the mutants. Among CD36 known ligands, lipids are candidate molecules, which could induce olfactory responses as environmental cues. In fact, we found that *Cd36*^*obl/obl*^ mice animals exhibited significantly decreased investigation time towards a lipid mixture containing saturated and unsaturated long-chain fatty acids (LCFAs), when compared to wild-type mice (Fig. 8c).

## Discussion

Previous studies have shown that CD36 and its homologue play a role in chemosensory responses to lipid ligands in insects and mammals^16^,^19^. In mice, CD36 is involved in gustatory detection of lipids, and in preferential attraction to lipid-enriched foods and beverages, responses which are abolished in CD36-null mice^19^,^30^,^31^.

Here we show that CD36 is highly expressed in a subset of mature OSNs present in the MOE. These neurons are located in the mature OSN layer and scattered in all zones of the olfactory epithelium. The number of CD36 expressing neurons increases during development, and persists in adult mice. These findings raise important questions regarding the possible functional roles played by CD36 in olfaction.

It has become clear now that the MOE is composed of distinct subpopulations of olfactory neurons. Some of these are well known, like the canonical olfactory sensory neurons, others have been only recently characterized; and there may be other subpopulations, yet to be identified. As mentioned above, the many subpopulations are mainly distinguished by the type of receptor they express: ORs, TAARs, GC-D, and there are also a few olfactory neurons that express V1Rs or V2Rs^32^,^33^. Here we identify a new subpopulation of olfactory sensory neurons which express CD36, a receptor that binds lipid ligands and has been previously proposed as a candidate fat taste transducer^34^.

Some olfactory neurons express members of the Trp channel family, however, it is not clear yet whether they work as receptors or simply as transducing effectors^4^. A newly identified population of *Trpc2*-expressing OSNs was recently reported, indicating that previous findings attributed to the VNO *Trpc2*-positive neurons could also be mediated by MOE cells^35^. Similarly, our results suggest that, in the olfactory system, the CD36 molecule may have similar functions attributed to these proteins in taste cells.

CD36-positive neurons coexpress *Omp* and *Gnal*, initially suggesting that they represent a subpopulation of canonical OSNs. We therefore analyzed whether these cells also express ORs. We did not detect coexpression of *Cd36* with 13 different tested ORs; however, we observed a frequent coexpression of *Cd36* with *Olfr287*. Since ~16% of the *Cd36*^+^ OSNs in OE zone 4 coexpress *Olfr287* we could predict, assuming equivalent proportions, that the remaining *Cd36*^+^-OSNs within this same expression zone would express at least additional five OR genes. Also, if *Cd36*^+^ OSNs in the other expression zones show similar coexpression frequencies as *Cd36*^+^/*Olfr287*^+^-OSNs, we would expect that additional eighteen ORs are coexpressed in these cells, totalizing ~24 different OR genes that can be coexpressed with CD36 in the whole OE. The observation that ~40% of *Olfr287* cells do not express *Cd36* suggests heterogeneity of these neurons. In fact, this is not an unpreceded finding, since Omura and Mombaerts^35^ showed recently that approximately a third, and not all, of *Olfr68*/*Olfr69*^+^ MOE cells express *Trpc2*. Altogether, it seems that some OSNs in the MOE are heterogeneous in terms of expression of accessory molecules, even if they have the same *Olfr* gene identity. Future experiments will be required to determine the complete set of ORs expressed in this subpopulation of OSNs.

Since CD36-OSNs do not represent a major subpopulation of OSNs, one would expect little impact in general olfactory parameters. The general morphology and organization of the CD36-deficient MOE is comparable to wild-type mice, which is also valid for general olfactory behavioral parameters. As mentioned above, CD36 in adipose and taste cells binds LCFAs^13^. In order to characterize specific olfactory behavior changes possibly related to CD36 loss of function, we tested whether CD36 deficiency impairs preference for a chemically defined lipid concentrate rich in LCFAs. We found that even though *Cd36*^*obl/obl*^ mice show normal general olfactory behaviors towards regular odorants, they do not show preference for a lipid mixture when compared to wild-type mice. Preference tests for lipid mixtures have been previously performed in other studies, but the specific contributions of the gustatory or olfactory systems to the behavioral responses could not be distinguished in these reported experiments^19^. Our study aimed to test specifically olfaction-driven instinctive behavioral responses. However, we cannot rule out the possibility that low concentrations of volatile fatty acids detected by CD36-taste cells contributed to the observed behavior. It is also possible that a generalized CD36-deficiency provokes specific impairment of lipid-elicited responses evaluated by preference tests, excluding an olfactory role for CD36. Additional experiments, including those involving OSN-specific *Cd36* deficient mice, are necessary in order to support the hypothesis that CD36^+^-OSNs are true lipid sensors functionally coupled to a brain circuitry involved in lipid preference behavior. Nevertheless, our results raise the interesting possibility that CD36 expressing olfactory neurons are involved in the recognition of lipid odorants, mediating the attraction to this type of chemosensory cue, especially because long-chain fatty acids should be present at very low concentrations in the air-phase. In this case, lipid-associated signal transduction mechanisms in the MOE could influence important social and feeding behaviors. For instance, the fatty alcohol (Z)-5-tetradecen-1-ol from exocrine glands was recently identified as a compound that enhance male urine attractiveness to female mice^36^. Recently, a study performed with human subjects suggested that fat content of food can be detected via odors^37^, implying that lipid odorants may be more physiologically relevant than previously assumed. One may expect that fatty odors would be important to identify highly caloric food that should be strategically consumed, but this hypothetical behavior may function in the opposite direction in a different metabolic context.

Previous studies have implicated CD36 in neuronal functions. For instance, it was shown that a subpopulation of glucose-responsive hypothalamic neurons senses fatty acids through CD36^38^,^39^. In addition, different studies implicated CD36 in lipid intake, satiety and obesity^40^,^41^,^42^,^43^. Although there is insufficient evidence to conclude that olfactory CD36 mediates fatty acid detection, the identification of a novel population of olfactory neurons expressing CD36 provides a new perspective to neuronal signaling with relevant impact to olfactory functions. It is known that each subpopulation of OSNs makes distinct connections in the OB, indicating that they provide information from different chemosensory inputs^27^. It will be interesting to determine how the axonal projection from CD36 expressing neurons to the main OB is organized. The fact that, unlike other OSN subpopulations, the CD36^+^-neurons are widely distributed all over the olfactory epithelium, and not segregated in different expression zones, suggests that the projection pattern of these neurons to the OB is different. It is possible that these neurons relay information in non-conventional forms that still need to be clarified.

Importantly, the possibility that CD36 may not work as a receptor involved in odorant signal transduction cannot be excluded. For example, CD36 could function as a metabolic sensor that informs the neurons about nutritional status or even transport fatty acids through the cell membrane. In addition, as an innate immune signaling co-receptor, CD36 can also sense lesions or nasal flora imbalance. These possibilities will have to be carefully addressed in order to determine the roles played by CD36 in olfactory physiology. During the preparation of the revised version of this manuscript two independent groups^44^,^45^ also reported expression of CD36 in OSNs and supporting cells.

## Methods

### Animal experimentation

*Cd36*^*obl*^ /Mmucd mice^15^ were acquired from MMRRC (UC Davis) in heterozygosis and were intercrossed to yield homozygotes mutants and wild-type mice; genotyping was performed by PCR followed by DNA sequencing. Wild-type C57BL/6J and *Olfr*^*17tm7Mom*^ /MomJ (P2-IRES-tauGFP) mice were obtained from the Jackson Laboratories. Animal experimentation was approved by the local Committees (COBEA-USP and *Comitê de Ética em Pesquisa* – UNIFESP) and the methods were carried out in accordance with the approved guidelines.

### Tissue dissection and total RNA extraction

Tissues were dissected from male mice. Dorsal tongue dissection was conducted by minimizing skeletal muscle contamination and prioritizing superficial epithelial tissue. Total RNA was extracted using Trizol^®^ reagent (Invitrogen™) according to the manufacturer instructions. Prior to cDNA synthesis we carried out DNAse I treatment (Qiagen^®^) on the RNA, followed by purification with RNeasy^®^ mini kit (Qiagen^®^).

### cDNA synthesis and qPCR

cDNA was synthesized from 1 μg of purified total RNA as described previously^46^. Briefly, 1 μg of total RNA and 100 ng of oligo (dT) were incubated for 2 minutes at 70 °C followed by rapid chilling on ice. cDNA was synthesized in the presence of RNAse inhibitor (RNaseOUT™, Invitrogen™) and 200 U of Superscript II reverse transcriptase (Invitrogen™) in 1x Superscript II first strand buffer at 42 °C for 60 minutes. *Cd36*, *Actb* and *Gapdh* transcripts were quantified by real-time PCR using the ABI 7300 Real-Time PCR system (Applied Byosystems^®^). Primer sequences for *Cd36* were GATGACGTGGCAAAGAACAG (forward) and TCCTCGGGGTCCTGAGTTAT (reverse)^19^. Primer sequences for *Actb* were GCAAGCAGGAGTACGATGAG (forward) and CCATGCCAATGTTGTCTCTT (reverse). Primer sequences for *Gapdh* were AAATGGTGAAGGTCGGTGTG (forward) and TGAAGGGGTCGTTGATGG (reverse). All reactions were performed by using a standard real time PCR protocol (1 cycle of 95 °C for 10 min, 40 cycles of 95 °C for 15 s, and 60 °C for 1 min). Data was normalized by using *Gapdh* and *Actb* as references. Relative gene expression across tissues was then calculated as 2^−ΔΔCt^ with the respective standard deviation values, using the OE sample as calibrator, according to Livak *et al*.^47^. Each reaction was performed in triplicate and the standard deviation was inferior to 0.3. *Actb* was preferred to *Hprt* as normalizer because the former showed less variability among the different tissues. Nevertheless, both normalizers produced similar results (Fig. S1).

### Tissue Preparation for Histology

Newborns were sacrificed by decapitation and heads were immediately fixed by immersion. The other mice were deeply anesthetized via an intraperitoneal injection of a mixture of ketamine hydrochloride and xylazine and then rapidly perfused transcardially with 0.9% saline, followed by 4% formaldehyde in Borax pH 9, at 4 °C. The noses were removed rapidly, postfixed and decalcified in 2% formaldehyde/0.25 M EDTA, and then placed in 20% sucrose solution overnight at 4 °C for cryoprotection. For fresh frozen sections the fixation step was skipped. The noses were embedded in Tissue-Tek^®^ O.C.T.™ media and were cut on a Microm cryostat (Thermo Scientific™) into 16 μm coronal sections. The slices were collected onto silane-coated adhesive microscope slides and stored at −20 °C.

### Immunofluorescence (IF)

The fresh frozen sections were fixed in cold methanol (−20 °C for 20 min), washed with PBS several times and incubated with blocking solution (PBS supplemented with 1% BSA and 4% donkey serum) followed by the addition anti-CD36 (EMD Millipore, #MAB1258) and anti-acetyl-α-tubulin (Lys40) (Cell Signaling Technologie^®^, # 5335S) antibodies. Subsequently, the sections were washed and primary antibody detection was conducted with compatible Alexa Fluor^®^ -488 and -546 secondary antibodies (Molecular Probes^®^). Conventional immunofluorescence in fixed tissue was performed by incubating washed sections in blocking solution (PBS containing 4% donkey serum, 1% BSA, and 0.4% Triton™ X-100) for 30 min. Using the same buffer solution composition, the sections were incubated over night at 4 °C with primary antibody anti-OMP (WAKO, Ltd. Japan #544–10001) or anti-TUBB3 (Aviva System Biology, CA #ARP63784). After incubation with the primary antibody, OE sections were rinsed in PBS and incubated with compatible Alexa Fluor^®^ -488 and -546 secondary antibodies for 120 min. Sections were then rinsed with PBS and counterstained with DAPI for nuclear labeling. Staining controls were performed to assure the specificity of CD36 labeling (Fig. S3).

### cRNA probe synthesis

*Cd36* sense and antisense riboprobes were synthesized as described before^20^, except that digoxigenin (DIG)-11-UTP (Roche) or DNP-11-UTP (PerkinElmer) were used instead of an isotopic labeled nucleotide. *Gnal* and *Omp* riboprobes were synthetized as described^48^, while *Taar7b*, *Cyp2a4, Olfr78*, *Olfr124*, *Olfr287*, *Olfr569*, *Olfr638*, *Olfr646*, *Olfr691*, *Olfr692*, *Olfr1264*, *Olfr1372-ps1, Olfr1507*, *Olfr1509* and *Olfr1512* DNA segments were amplified by PCR from OE cDNA. Primers used for *Taar7b* were CTACCCCACCAGGTTTACT (forward) and AGGAATTCTCCCTCAAGACT (reverse); primers for *Cyp2a4* were AGAGATTTGCAGACCTGATCC (forward) and ATGCAGCCCAGGATCAACG (reverse); primers for *Olfr1507* were TTTTAAATTGTCCTGACAAACTGG (forward) and TCTGATTCTCTCAGTCCCTTCA (reverse); primers for *Olfr78* were GAGGAAGCTCACTTTTGGTTTGG (forward) and CAGCTTCAATGTCCTTGTCACAG (reverse); primers for *Olfr124* were GGTAATATCTCCATTATCCTAGTTTCCC (forward) and TTGACCCAAAACTCCTTTGTTAGTG (reverse); primers for *Olfr287* were GGGCTCTTGGGATAGGTAG (forward) and GGGCTGGCTTACAGGTTTAG (reverse); primers for *Olfr569* were TAACAGCTCTTCCCATCCCCTGTTC (forward) and TAGGGTTGAGCATGGGAGGAACAAGC (reverse); primers for *Olfr638* were AGAAGTAACTAACACCACTCATGGC (forward) and TTAGTGCACCTTTCTTTGCAAC (reverse); primers for *Olfr646* were ATGAGCGAAAGTCTCCCAGTCACTC (forward) and AAGTGAAGCAGTTTTAGAAGCCGAC (reverse); primers for *Olfr691* were TGGGTTGGAGGCTTATCATACCTG (forward) and AAGAACAACACAGAGTCTTGATGTC (reverse); primers for *Olfr692* were AAACTTCATCCTTACAGAATGGCAG (forward) and ACTGGCTTTGGGACAGTGTGA (reverse); primers for *Olfr1264* were TTTCTCAGAGGCCAGAGATCCAG (forward) and CTACAACTTCTGAGCGTGTGAAGAG (reverse); primers for *Olfr1372-ps1* were CCCTAGGTCATTTAGCAGTTATTGTCAC (forward) and CAGATCTTGACTTGATCTTCAACACTG (reverse); primers for *Olfr1509* were ATGGGAGCTCTAAATCAAACAAGAG (forward) and TAGAAAACCGATACCACCTTGTCG (reverse); primers for *Olfr1512* were TACATCCTGACTCAGCTGGGGAACG (forward) and GGGCACATAGTACACAGTAACAATAGTC (reverse).

### *In situ* hibridization, two-color fluorescence *in situ* hybridization (FISH) and double-labeling ISH/IF

Pre-hybridization/hybridization was conducted according to an adapted protocol from a previously described procedure^49^. Briefly, the OE sections were immersed in 4% formaldehyde, followed proteinase K treatment at 37 °C and acetylation in triethanolamine solution. Tissue sections were dehydrated through graded concentrations of ethanol and air-dried. Hybridization solution containing the probe was spotted onto the slides, which were coverslipped and incubated overnight at 60 °C in a slide moat incubator (Boekel; Feasterville, PA). Post-hybridization was carried out with several washes in decreasing concentrations of standard saline citrate (SSC) solution and RNAse treatment. Chromogenic DIG detection was performed according to manufacturer by employing alkaline phosphatese (AP)-conjugated antibodies against DIG and NBT/BCIP as substrate (Roche). Two-color FISH was performed adapting a previously described protocol^50^. DIG-labeled probes signals were developed with tyramide-Alexa Fluor^®^ 488 (Tyramide signal amplification kit, Molecular Probes^®^), which was followed by HRP quenching with 3% H_2_O_2_, several washes and anti-rabbit-HRP (Jackson ImmunoResearch; West Grove, PA) incubation. DNP-labeled probes signals were developed with tyramide-Alexa Fluor^®^ 555 (Tyramide signal amplification kit, Molecular Probes^®^). In some assays fluorophore labeling for each hapten detection was inverted, and we also made use of tyramide-Alexa Fluor^®^ 546, and tyramide-biotin (PerkinElmer)/Avidin- Alexa Fluor^®^ 488 reagents (Molecular Probes^®^) along with Avidin/Biotin Blocking kit (Vector Laboratories). For ISH/IF combination, IF was performed after ISH signal development using a shorter proteinase K treatment protocol.

### Olfactory behavioral assays

Risk assessment behavior exhibited towards predator odors was used as a VNO-mediated behavior in our control investigation of the effect of CD36 genetic ablation on VNO function. Risk assessment behaviors have been consistently used as indicators of defensive behavior in many animals^51^. The experiments were performed as previously described^29^. Animals were habituated in the procedure room for two consecutive days, in the dark, for 90 min each time. On the following day, mice were assayed by exposing them to a control clean sterile and deodorized medical gauze, or to a medical gauze rubbed against the fur of a domestic cat. Each exposure session was filmed and the digitalized movies were scored blindly to annotate risk assessment episodes, which were quantified for the first 12 min of the assay.

The buried food test was carried out based on protocols described in previous studies^52^. Mice were placed in home cages (polysulfone, 46 cm L × 23.5 cm W × 20 cm H) with 5 cm of clean bedding and subjected to fasting starting on the afternoon on the day before the behavioral assays. Mice were manipulated and allocated into the experimental room for one hour habituation 48 and 24 h prior the test day, and were fasted for 24 hours before the test. The experiment was performed placing each mouse, individually, in the middle of a clean cage containing 5 cm deep of clean bedding and the stimulus (food pellet measuring about 1 cm) was hidden at the bottom of the cage, in a random corner of the cage. We considered that the animal found the food pellet when it started eating it, usually holding the pellet with its forepaws. Each experimental trial lasted for 5 minutes; therefore, the maximum score was 300 sec. After the test, mice returned to their home cage and food was replaced *ad libitum*.

The innate odor preference test was conducted as previously described^53^. In these experiments, mice were used only once in order to avoid adding the confounding factor of olfactory learning. Mice (n = 5–10) were individually caged (test cages were 50 L × 30 W × 25 H cm) two days prior to the onset of each experimental trial. They were then habituated for 30 min four times on two consecutive days by placing them on a clean cage identical to the home cage, but without bedding, food grid or water bottle. On the test day, mice were transferred to the empty test cage with a deodorized filter paper (2 × 2 cm) scented with PBS (control), amyl acetate (1 mM), or a chemically defined lipid concentrate (Gibco^®^; lipid composition in (mg/l): arachidonic acid (2.0), cholesterol (220.0), DL-alpha-tocopherol acetate (70.0), linoleic acid (10.0), linolenic acid (10.0), myristic acid (10.0), oleic acid (10.0), palmitic acid (10.0), palmitoleic acid (10.0), and stearic acid (10.0)). Similar results were obtained when the concentration of amyl acetate used was 100 μM (not shown). In each trial, the filter paper was placed inside a metallic wire ball (tea ball) to prevent the animals from directly contacting the paper or probing it with the tongue. Behaviors were recorded on digital video camera and investigation times were measured for a 5 min exposure session.

### Image processing

Bright-field and epifluorescence photomicrographs (Nikon^®^ or Carl Zeiss) or confocal acquisitions (Carl Zeiss LSM 780 and Leica SP8) were processed to enhance contrast and image quality using GIMP (http://www.gimp.org/) and were assembled using Inkscape (http://inkscape.org). Pictures representing different groups received equivalent image treatment. Orthogonal projections from confocal acquisitions were exported from BioImageXD free analysis software (www.bioimagexd.net).

### Quantitative analysis

During postnatal development, the density of *Cd36*-positive cells in OSN layer were determined by counting the total number of completely stained nucleated cells per linear length of the OE (lamina basal as reference) in each field (region of interest), or per OE area. It is important to point out that robustly stained *Cd36* cells in OSN layer, contrast with the weakly stained supporting cells, since signal development was controlled. Transcript coexpression in the same cells was carefully conducted both in widefield and confocal microscope acquisitions. We only considered for counting transcript-positive cells in the OSN layer with robust staining, defined nucleus, and restricting *Cd36*^+^-OSNs counting to the same OE zone where the ORs are expressed. In some few cases, coexpression could not be determined and was classified as “unsure”.

### Statistical analysis

A student’s t-test variation, Welch’s test, was employed for comparison between two groups according to homogeneity of variances and normality tests. For more groups and two grouping categorical independent variables, comparison of means was performed using a two-way analysis of variance (ANOVA) followed by a Tukey’s HSD procedure as post hoc multiple comparisons test when pertinent. Frequencies were evaluated with binomial test (expected frequencies) or Fisher’s exact test (contingency tables). The analyses were carried out with R (cran.r-project.org; base, and packages RVAideMemoire, car and phia). Heatmap was generated using ordered rows imposed by dendrogram according to the function heatmap.2 (gplots; R). Co-localization probability was calculated with BioImageXD (www.bioimagexd.net) according to Costes methods^54^.

## Additional Information

**How to cite this article**: Xavier, A. M. *et al*. CD36 is expressed in a defined subpopulation of neurons in the olfactory epithelium. *Sci. Rep*. **6**, 25507; doi: 10.1038/srep25507 (2016).

## Supplementary Material

Supplementary Information

## Figures and Tables

**Table 1:** CD36 coexpression with mature OSN markers.

Transcript or Protein	*Cd36* (mRNA)-positive cells			
	*n* of cells negative for coexpression	*n* of cells positive for coexpression (%)	*n* of cells uncertain for coexpression	Total *Cd36*^+^ cells counted
*Omp*	–	107 (92%)	10	117
*Gnal*	4	98 (93%)	7	105
*Taar7b*	68	−(0%)^†^	–	312
*Olfr1507*	3321	−(0%)^#†^	–	3209
Tau-EGFP (P2)	94	−(0%)^†^	–	366
OR pool 1	137	−(0%)^†^	–	191
OR pool 2	829	−(0%)^#†^	–	1471
OR pool 3	615	−(0%)^#†^	–	688
*Olfr287*	40	65 (16%)*	–	407

The numbers of *Cd36*^+^-OSNs coexpressing the different mature OSN markers are shown. Cell counting was performed for each pair of transcripts using the same microscope fields and restricting *Cd36*^+^-OSNs counting to the same OE zone where the ORs are expressed. We only considered for the analysis cells showing defined nuclei. Stained supporting cells were excluded from the analysis. Pool 1: *Olfr124* and *Olfr1509*; pool 2: *Olfr646*, *Olfr1264* and *Olfr1372-ps1*; pool 3: *Olfr78*, *Olfr569*, *Olfr638*, *Olfr691*, *Olfr692*, and *Olfr1512*). ^#^Significantly smaller than [0.1% x (number of OR genes per probe)] (p < 0.05); *Significantly different than 0.1% (p < 2.2 × 10^−16^); ^†^Significantly different from coexpression frequency for *Olfr287* (p < 1 × 10^−10^; Bonferroni correction).
