# Supplementary material for: CD36 is expressed in a defined subpopulation of neurons in the olfactory epithelium
Source: Sci Rep. 2016 May 5;6:25507. doi: 10.1038/srep25507 (PMC4857125; doi:10.1038/srep25507)
Supplement: Supplementary Information [file srep25507-s1.pdf]

CD36 is expressed in a defined subpopulation of neurons in the olfactory epithelium

André Machado Xavier[1], Raissa Guimarães Ludwig[1], Maíra Harume Nagai[2], Tiago Jonas de Almeida[2], Hebe Mizuno Watanabe[2], Marcio Yukio Hirata[3], Tatiana Rosado Rosenstock[4], Fabio Papes[3], Bettina Malnic[2] and Isaias Glezer[1*]

[1] Department of Biochemistry, Escola Paulista de Medicina, Universidade Federal de São Paulo
[2] Department of Biochemistry, Instituto de Química, Universidade de São Paulo

[3] Department of Genetics and Evolution, Instituto de Biologia, Universidade de Campinas
[4] Department of Physiological Science, Santa Casa de São Paulo Medical School

All correspondence to: Dr. Isaias Glezer
Tel: (+5511) 5576-4445
Fax: (+5511) 5572-3006
Email: iglezer@unifesp.br

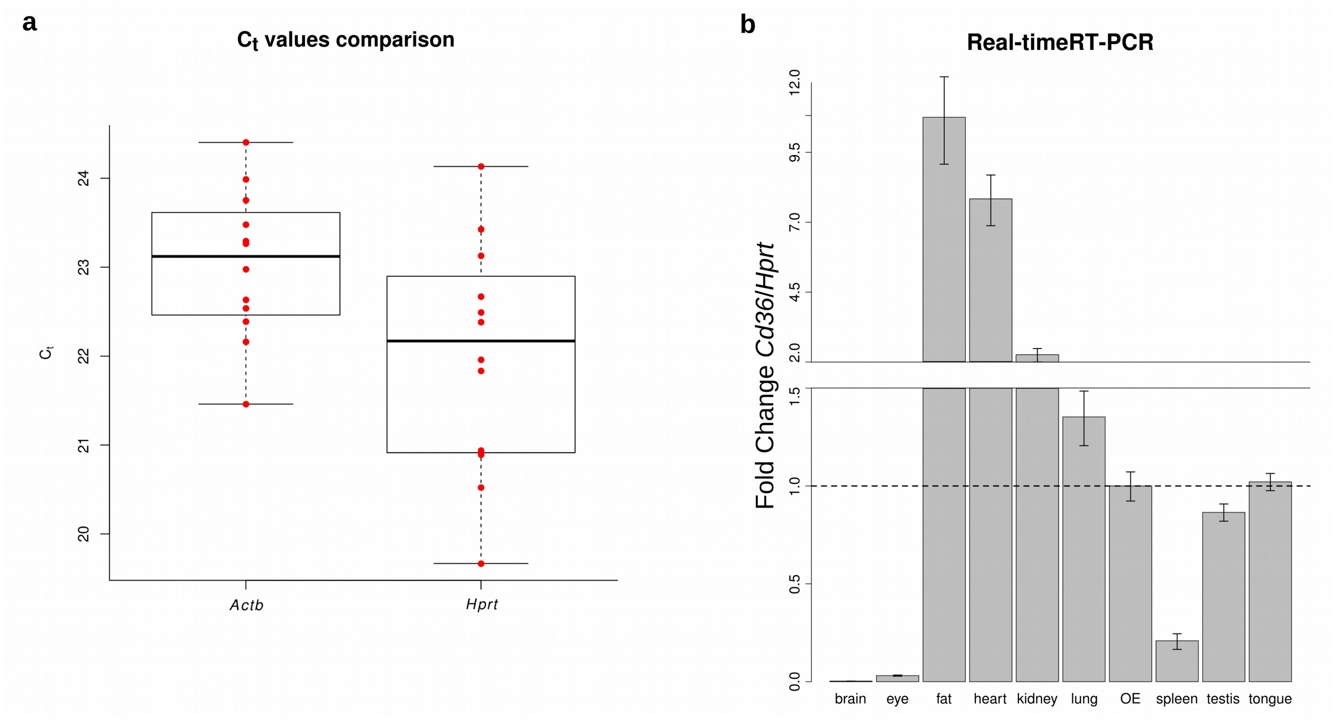

Figure S1 - *Cd36* mRNA expression levels in the olfactory epithelium and different tissues of mouse using the housekeeping gene *Hprt* as normalizer. (**a**) Comparison of C_t values for *Actb* and *Hprt* extracted from different tissues. Normalizers should vary as minimally as possible. (**b**) Real-time RT-PCR analysis of *Cd36* transcript levels expressed as fold-change and normalized against *Hprt* for each tissue (OE as calibrator).

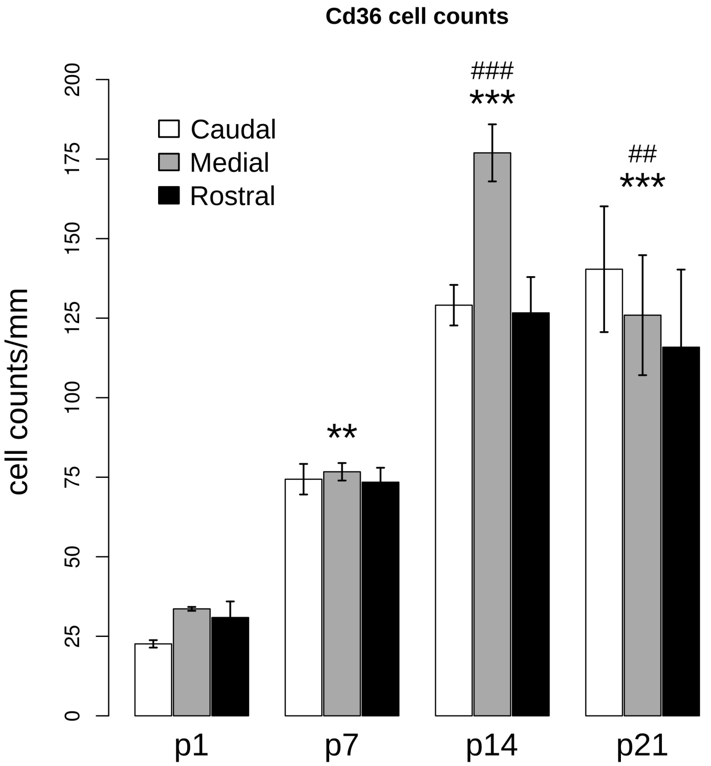

Figure S2 - Cellular counts of *Cd36* mRNA-positive cells during postnatal development of the olfactory epithelium corrected for the linear extension of the epithelium. Quantification of cell density (cell counts/OE linear length; mean ± S.E.M.), which was performed only considering strong labeled cells in the MOE and excluding the stained apical contour cells (supporting cells; Scs). Two-way ANOVA revealed a highly significant main age effect $F_{(3,24)} = 59.168$, $p = 3.1 \times 10^{-11}$, while the level of the olfactory epithelium showed no main effect, $F_{(2,24)} = 2.090$, $p = 0.146$; interaction was absent ($p = 0.169$). The p values for each comparison were: $p<0.0005$ (**) against p1; $p<0.00001$ (***, ###) against p1 and p7, respectively and $p<0.0001$ (##) against p7.

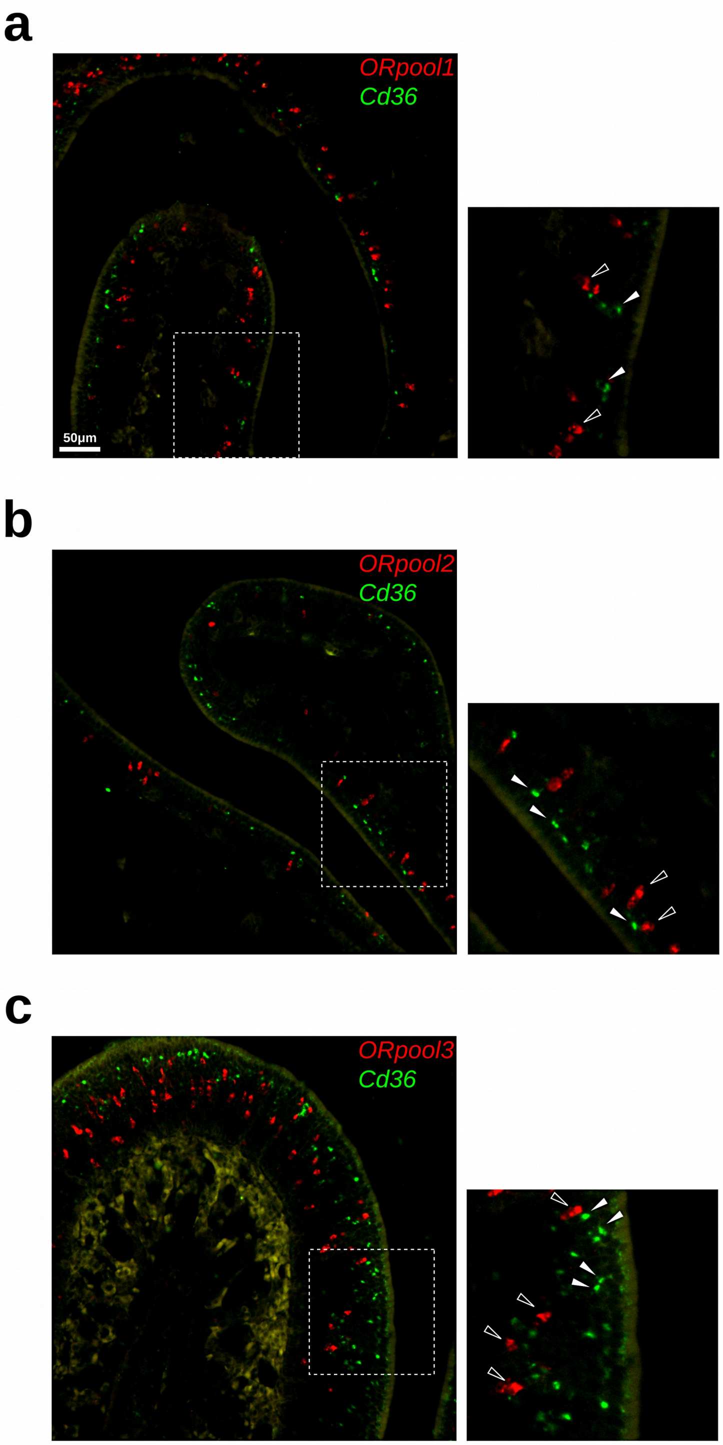

Figure S3 - *Cd36* expressing neurons are not frequently associated with varied OR genes. (**a-c**) Two-color fluorescent in situ hybridization (FISH) with a *Cd36* riboprobe shown in green (white arrowheads) and the other transcripts shown in red (empty arrowheads). No *Cd36* coexpression was observed for three different mixtures of ORs probes comprising: (**a**) pool 1: *Olfr124* and *Olfr1509*; (**b**) pool 2: *Olfr646*, *Olfr1264* and *Olfr1372-ps1*; and (**c**) pool 3: *Olfr78*, *Olfr569*, *Olfr638*, *Olfr691*, *Olfr692*, and *Olfr1512*. Please refer to table 1 in the main text for quantitative details. Scale bars are shown.

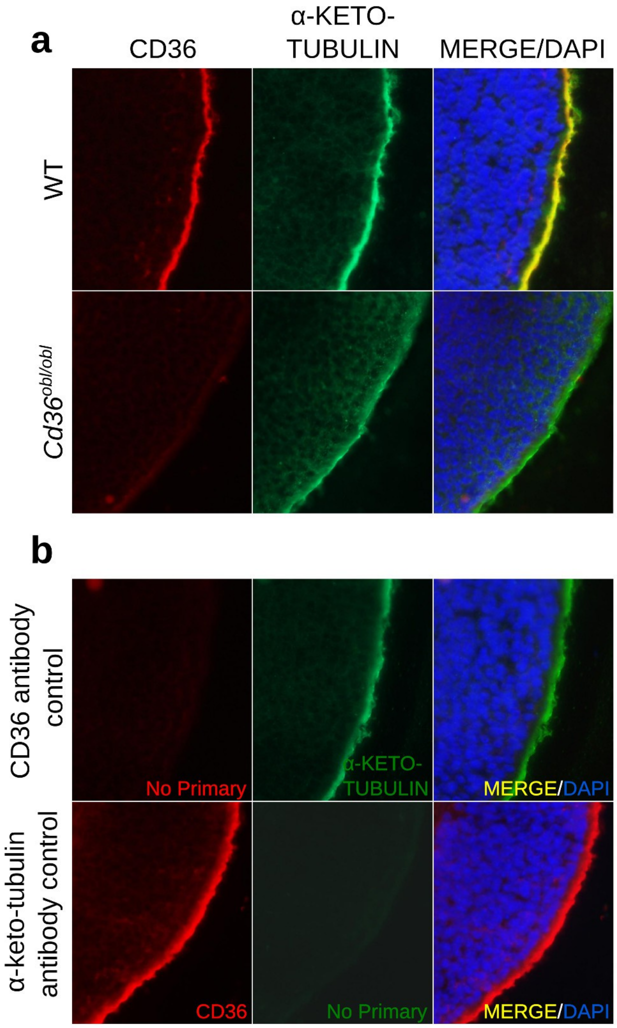

Figure S4 - CD36 antibody specificity in the immunofluorescence assay. Fluorescence microscope images of olfactory epithelium sections from wild type and *Cd36*$^{obl/obl}$ mutant mice immunostained with antibodies against CD36 (red) and acetylated α–tubulin (green, α-KETO-TUBULIN). No staining for CD36 is observed in the OE from *Cd36*$^{obl/obl}$ mice (**a**); (**b**) Omission of primary antibodies (negative control).

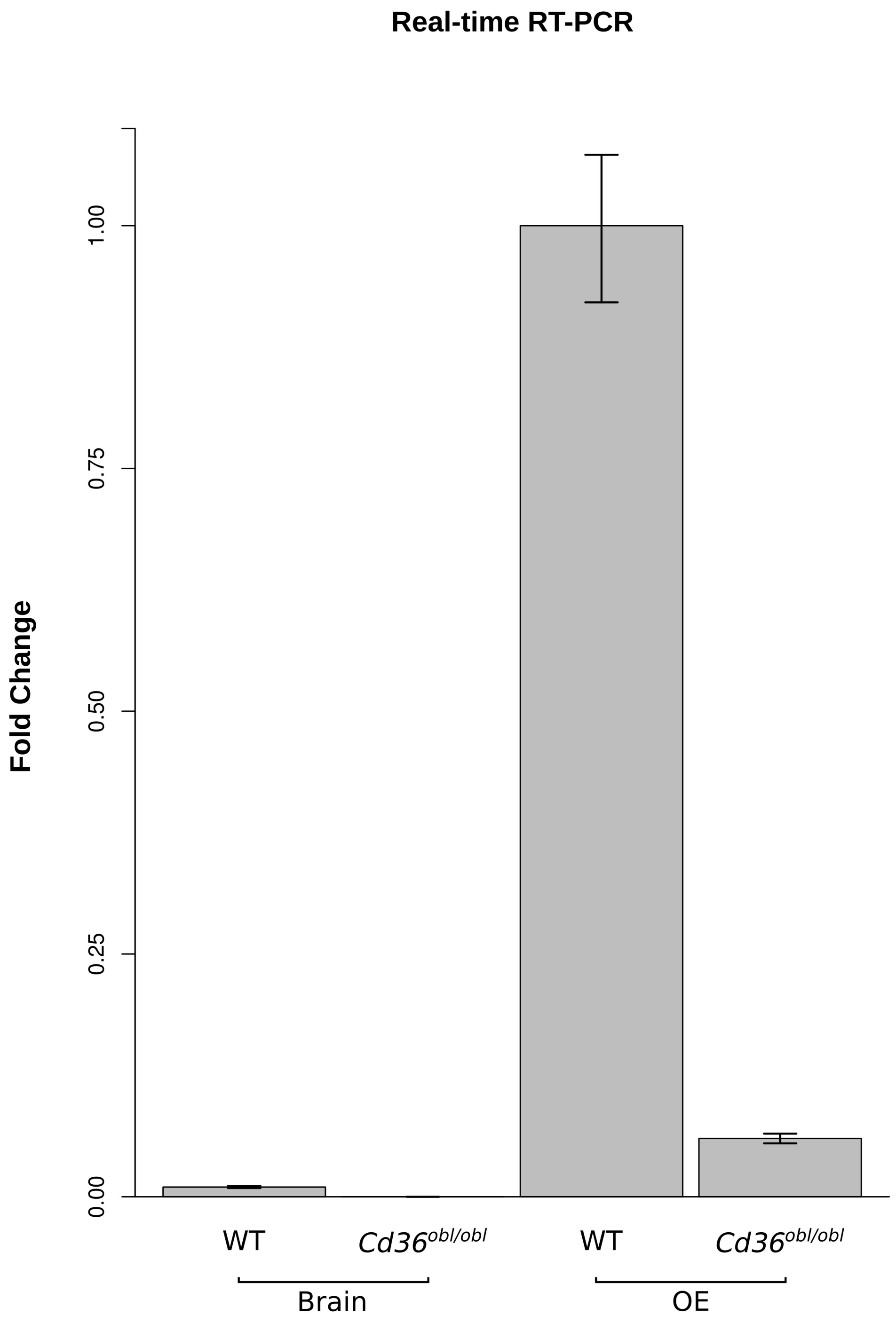

Figure S5 - Decreased *Cd36* mRNA levels in the *Cd36*$^{obl/obl}$ mutant strain. The levels of *Cd36* transcripts in the olfactory epithelium and brain from C57Bl/6 and *Cd36*$^{obl/obl}$ mice were determined using real-time RT-PCR and *Actb* as normalizer. The assay revealed an expressive reduction of transcript levels in *Cd36*$^{obl/obl}$ mouse in both tissues analyzed when compared to wild-type animals.

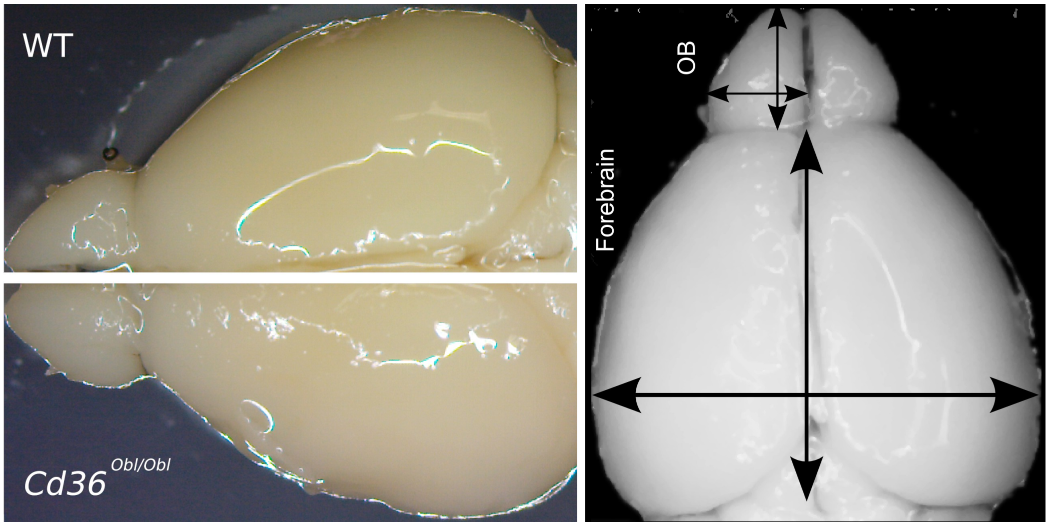

Figure S6 – **Absence of gross anatomical changes in one-year-old CD36-deficient mice OB**. Photographs from formaldehyde-preserved brain depicting representative OB and forebrains from wild-type (WT) and *Cd36*$^{Obl/Obl}$ mice. At left, longitudinal (anterior-posterior) and lateral measurements, according to a maximum distance for the respective plane. Please refer to table S1 for measurements.

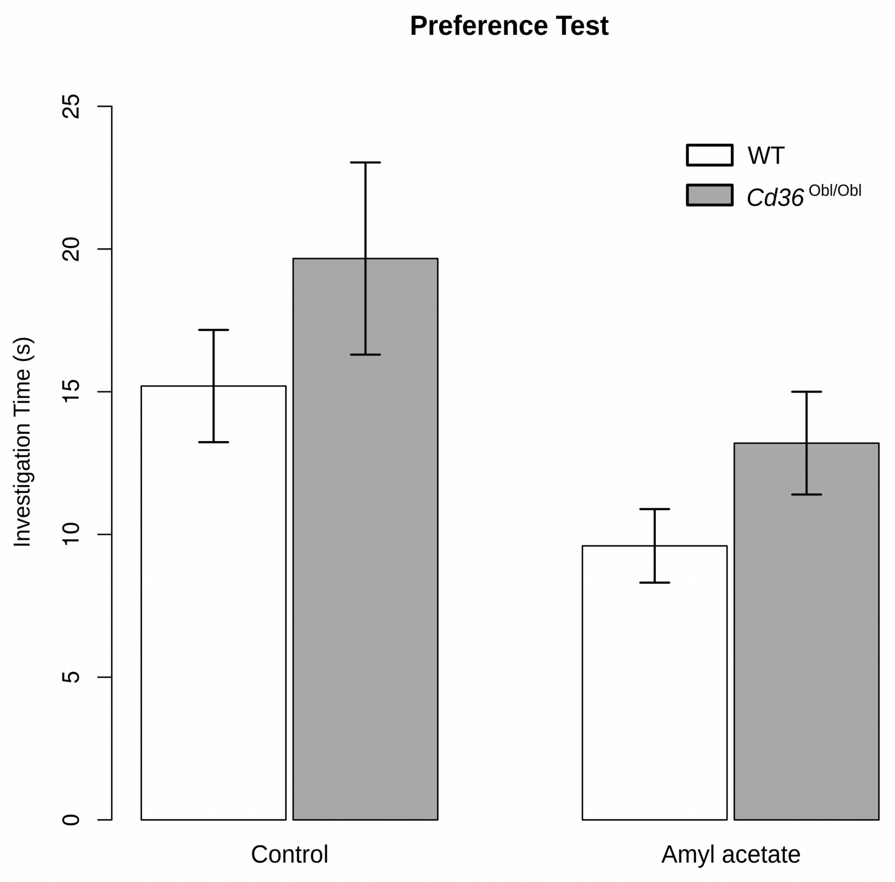

Figure S7 – CD36-deficient mice do not display altered responses to a neutral odorant in an olfactory preference test. Wild type and *Cd36*$^{obl/obl}$ mice were exposed to scented filter papers after habituation and the investigation time was recorded. Amyl acetate, considered to be a neutral odorant, was used as stimulus. No significant difference was observed between the two strains (*n* = 6 in controls and *n* = 5 for amyl acetate exposed groups).

Genotype	OB			
	Longitudinal (mm)		Lateral (mm)	
	Mean	SD	Mean	SD
WT	2.759	0.291	2.184	0.081
Cd36$^{Obl/Obl}$	2.708	0.186	2.141	0.057
	**Forebrain**			
	Longitudinal (mm)		Lateral (mm)	
	Mean	SD	Mean	SD
WT	8.700	0.200	10.18	0.144
Cd36$^{Obl/Obl}$	8.681	0.354	10.12	0.107
	**Ratio OB/forebrain**			
	Longitudinal		Lateral	
	Mean	SD	Mean	SD
WT	0.3176	0.038	0.2146	0.006
Cd36$^{Obl/Obl}$	0.3120	0.018	0.2116	0.004

Table S1 – Macroscopic measurements from one-year-old wild-type (WT; *n* = 6) and CD36-deficient mice (*n* = 4). Photographs from formaldehyde-preserved brain were acquired using a digital camera connected to a stereo microscope (Zeiss) and subsequently analyzed with Image J software in order to perform measurements. The longest parasagittal and transversal distances were considered for longitudinal and lateral dimension determination, respectively, as depicted in the scheme in figure S6. No statistically significant differences were observed between the genotypes (Welch's test), which was also the case when considering OB/forebrain ratios for each individual.
